# How Does the Interaction Between Preterm Delivery and Low Birthweight Contribute to Racial Disparity in Infant Mortality in the United States?

**DOI:** 10.3390/jcm14186422

**Published:** 2025-09-11

**Authors:** James Thompson

**Affiliations:** College of Veterinary Medicine and Biomedical Science, Texas A&M University, College Station, TX 77843-4475, USA; jthompson@cvm.tamu.edu

**Keywords:** causal inference, Bayesian, mediation modeling, infant mortality, potential outcome

## Abstract

**Background/Objectives**: In the United States, Black infants are twice as likely as infants of all other races and ethnicities to die by one year of age. Mediation modeling predicted that preventing low birthweight could alleviate 75% of this disparity. However, the potential confounding and interacting role of preterm birth remains a question. The goal of this study was to determine how birthweight and length of gestation interact in causing racial disparity. **Methods**: Records from more than 25 million singleton births were retrieved from the United States National Natality Database for the years 2016 to 2022. Two interaction models were evaluated using Bayesian estimation of potential outcomes. The first modeled the interaction between birthweight and length of gestation with both mediators measured as binary (normal/abnormal). The second modeled the interaction using five classifications for both birthweight and length of gestation. **Results**: Eliminating either abnormal birthweights or abnormal lengths of gestation would reduce racial disparity in infant mortality by approximately 75%. There was no additional reduction of racial disparity by normalizing both. Modeling the combinations of specific categories of birthweight and length of gestation showed Black infants were 2.76 (2.72, 2.79) times more likely to be born with extremely low birthweight and extremely preterm delivery. This single combination explained over 60% of the racial disparity in infant mortality. **Conclusions**: The current study clarifies how birthweight and preterm birth contribute to racial disparity and illustrates how Bayesian estimation of potential outcomes enables complex mediational investigations.

## 1. Introduction

Infant mortality (IM), defined as infant death before one year of age, is one of the deadliest racial health disparities [1,2]. In the United States (U.S.), the risk for Black infants, for the years 2016 to 2018, was double that for infants of all other races and ethnicities combined [3]. Despite considerable speculation, the causes remain largely uncertain [1,2]. Mediation modeling recently predicted that if low birthweight (BW) could have been prevented, nearly 75% of the racial disparity in infant mortality could have been alleviated [3]. The mediation study was conducted using a Rubin causal modeling (RCM) approach. The RCM approach provides causal inferences as conditional probabilities referred to as potential outcomes (POs) [4]. The racial disparity was also a causal inference and was estimated as the difference between POs for two race groups. The outcome-based RCM model has similarities to model-based approaches but also has differences that require distinct technical language to differentiate the two approaches [5]. Substantive among the differences is the approach to the identification strategy for causal effects. In the model-based approach, the identification strategy starts with a graphical model like a Directed Acyclic Graph (DAG) [5]. Rubin objected to identifying causal effects in a DAG, preferring specific identification and estimation [4]. This can be an important distinction. However, in the previous model [3] and in the proposed extension of this model, the causal effects can be identified by a DAG that provides a node to identify an interaction [6]. This interaction DAG provides arcs for every direct causal effect without there being any indirect causes [3,6]. In contrast, “causal identification” is an important issue in model-based approaches because indirect effects are not specified in a DAG. In model-based approaches, estimating proportions of direct and indirect effects is often referred to as decomposition [7]. There is no such decomposition in the RCM approach. A second substantive difference in terminology involves the term “counterfactual”. The model-based approach for a binary outcome, like IM, identifies a factual outcome for everyone, in the study, usually indicated by 0 or 1. The counterfactual outcome is a hypothetical one in which a potentially alternative outcome, also represented by 0 or 1, could have occurred had the risk factors been different. The outcome-based approach does not compare factual and counterfactual outcomes. The Bayesian approach estimates the probability of outcome = 1 for every individual in the sample as the potential outcome, assuming all individuals with the same vector of covariates had the same estimable but uncertain risk. It is the individual’s PO, not individual’s factual and counterfactual outcomes, that provide the bases for causal modeling. Use of the term “counterfactual” can be found in reports of outcome-based modeling but cannot mean the same as “counterfactual” in model-based causal modeling. For outcome-based modeling, “potential outcome” is the preferred technical term rather than “counterfactual estimates” [4].

The National U.S. Natality database records five BW categories: extremely low birthweight (ELBW), very low birthweight (VLBW), low birthweight (LBW), normal birthweight (NBW), and increased birthweight (also called macrosomia; MAC). The previous study [3] estimated the full mediational potential for optimizing BW and used a binary classification. Subsequent investigations should identify any information lost by the reduction of categories. The database also records length of gestation (LG) based on five categories: extremely preterm delivery (EPTD), very preterm delivery (VPTD), preterm delivery (PTD), term delivery (TD), and post-term delivery (PostTD). Although some combinations of BW and LG do not occur, a model should have the potential to estimate interaction effects with two levels of race and 25 levels of potential mediation.

The goal of the present study was to determine how BW and LG interact in causing racial disparity in infant mortality. The first objective was to estimate the percentage of racial disparity attributable to abnormal BW, abnormal LG, or a combination of both. This involved binary classification schemes of normal and abnormal. The second objective was to parse the overall effect, predicted in the first objective, and estimate the percentage of racial disparity attributable to specific combinations of abnormal BW and LG.

## 2. Materials and Method

### 2.1. Data

The source of data was the National Vital Statistics System which provides the data online (https://www.cdc.gov/nchs/nvss/births.htm, accessed on 15 January 2025). Data for the study are publicly available with all patient identifiers removed. The Texas A&M Institutional Review Board reviewed the protocol and deemed the study as not human subject research (NHSR). The NHSR status exempted the study from patient consent. The retrieved data included eight years of birth data (1 January 2016 to 31 December 2022) and nine years of infant mortality data (1 January 2016 to 31 December 2023).

### 2.2. Model

Infant mortality was defined as death before one year of age. Infant race (R) was the factor of interest and was coded as “Black” when the mother’s race was identified as Black and coded as “non-Black” for all other races. The Black race group included non-Hispanic black infants, Hispanic black infants, non-Hispanic black infants with multiple races, and black infants with unknown race. The potential mediators were BW and LG. Data missing BW or LG were deleted. In objective 1, BW was defined as normal for the range of 2.5 kg to 4.0 kg. All other BWs were defined as abnormal. LG was defined as normal for 37 weeks to 41 weeks. All other LGs were defined as abnormal.

Model 1 evaluated the interaction between BW and LG using binary classifications of both mediators and estimated rates for potential outcomes (POs) using direct Bayesian estimation. Model 2 evaluated the interaction between BW and LG using five classifications for both mediators. Mediators were classified using the following definitions for BW: extremely low BW (ELBW; <1.0 kg), very low BW (VLBW; 1.0 kg to 1.5 kg), low BW (LBW; 1.5 kg to 2.5 kg), normal BW (NBW; 2.5 kg to 4.0 kg), and macrosomia (MAC; >4.0 kg). The following definitions were used to classify LG: extremely preterm delivery (EPTD; <28 weeks), very preterm delivery (VPTD; 28 to 32 weeks), preterm delivery (PTD; 32 to 37 weeks), term delivery (TD; 37 to 41 weeks), and post-term delivery (PostTD; >41 weeks). The model for objective 2 estimated rates for POs using direct Bayesian estimation on data and a binomial prior. Data were aggregated into rows, with each row identified by unique values for race, BW, and LG. For i in 50 rows of data, the occurrences of infant mortality were counted as r [Race = 1:2, BW = 1:5, LG = 1:5] and the number of births as n [Race = 1:2, BW = 1:5, LG = 1:5]. Rows that had no observations were given values of zero for n and r. In the following equations, race is indexed R [1:2], BW is indexed BW [1:5], and LG is indexed LG [1:5]. The counts of deaths r [R_1:2_, BW_1:5_, LG_1:5_] were modeled as binomial with a rate parameter PO [R_1:2_, BW_1:5_, LG_1:5_] and count of births n [R_1:2_, BW_1:5_, LG_1:5_].

r [R_1:2_, BW_1:5_, LG_1:5_]~Binomial(PO [R_1:2_, BW_1:5_, LG_1:5_}, n [R_1:2_, BW_1:5_, LG_1:5_]). (1)

The rate parameters are PO estimates and were given beta(1,10) priors.

PO [R_1:2_, BW_1:5_, LG_1:5_] ~ beta(1,10). (2)

The standard definition was used for total effect (TE):

Prob(IM)|Race = 2 − Prob(IM)|Race = 1,(3)

and TE was estimated in the model by the racial difference in PO ignoring BW and LG.

TE = PO [R = 2] − PO [R = 1].(4)

The controlled direct effect (CDE) was defined as the difference between population race rates for infant mortality under a control strategy. A single control strategy of altering both BW and LG to normal was used in the evaluation. Controlling a risk was defined as modifying the PO for both race groups (Black, non-Black) to the PO observed when BW and LG were both normal. The full posterior distribution of expected cases in the two race strata (new count of deaths in strata; s.r [R_1:2_, BW_1:5_, LG_1:5_]) was estimated as follows:

s.r [R_1:2_, BW_1:5_, LG_1:5_] = (n [R_1:2_, BW_1:5_, LG_1:5_] * PO[R_1:2_, BW = normal, LG = normal]). (5)

This predicted modification would change the total population counts for infant mortality for both races. The full distribution of the predicted count of deaths in the population count p.r [R_1:2_, BW_1:5_, LG_1:5_] was estimated as the previous population count by race minus the change in the target stratum, by race:

p.r [R_1:2_, BW_1:5_, LG_1:5_] = r [R_1:2_] − (r [R_1:2_, BW_1:5_, LG_1:5_] − s.r [R_1:2_, BW_1:5_, LG_1:5_]). (6)

Full posterior distributions of the new projected POs (new.PO [R_1:2_, BW_1:5_, LG_1:5_]) were estimated by dividing the full posterior of a new count for infant mortality (p.r [R_1:2_, BW_1:5_, LG_1:5_]) by count of births by race (n [R_1:2_]):

new.PO [R_1:2_, BW_1:5_, LG_1:5_] = p.r [R_1:2_, BW_1:5_, LG_1:5_]/n [R_1:2_]. (7)

The CDE for changing the interacting risks defined for BW and for LG to normal for each was estimated as follows:

CDE [BW_1:5_, LG_1:5_] = new.PO [2, BW_1:5_, LG_1:5_] − new.PO [1, BW_1:5_, LG_1:5_]. (8)

Percentage attributable (PA) was estimated as follows:

PA [BW_1:5_, LG_1:5_] = 100 × (TE − CDE [BW_1:5_, LG_1:5_])/TE. (9)

Sensitivity to prior values was evaluated by repeating Model 2 using uniform(0,1) priors for PO.

All Bayesian modeling used Multibugs 1.0 [8]. A burn-in of 5000 iterations was discarded, and the next 10,000 iterations were collected for posterior distributions. Convergence was determined by monitoring chains with disparate starting values. The most disparate starting values possible were utilized. This included both 0 and 1 for rate parameters of binomial distributions and, for the count parameter (r), in a binomial distribution with n observations, the disparate starting values were r = 0 and r = n. Reported results are the median and 95% credibility interval, which were the 2.5 and 97.5 percentiles, respectively, taken directly from the posterior distribution. The MultiBUGS code and data are available in Appendix A.

## 3. Results

The records identified 25,475,195 singleton births, for seven years, from 1 January 2016 to 31 December 2022. Deleted observations included 14,222 (<0.1%) missing BW and 12,756 (<0.1%) missing LG, leaving 25,448,217 observations for analysis.

Visual observation of pairs of Markov chains showed immediate convergence for all parameters in all models.

There were 2,475,161 births of Black infants with 21,881 infant mortalities, with a rate of infant mortality (PO) for Black infants of 0.0088 (0.0087, 0.0090). Of black births, there were 2,126,358 non-Hispanic black infants, 227,017 Hispanic black infants, 10,501 non-Hispanic black infants with multiple races, and 18,285 black infants with unknown race. There were 22,973,056 births of non-Black infants with 101,641 infant deaths and a rate of infant mortality (PO) of 0.0044 (0.0042, 0.0043). The total effect for racial disparity was 0.0044 (0.0043, 0.0045), and the relative risk was 2.00 (1.97, 2.03).

For Model 1, the percentage of racial disparity attributable to abnormal BW was 74.6 (72.7, 76.4), to abnormal LG, 71.7 (69.7, 73.6), and to a combination of both abnormal BW and LG, 74.9 (73.0, 76.8) (Table 1).

For Model 2, two combinations of birth conditions, MAC/EPTD and MAC/VPTD, were not observed for either race classification. For these four POs, the posterior distribution of PO was identical to the beta(1,10) prior; these estimates did not contribute to other estimates reported here and are not reported. There were several combinations with fewer than 100 births. For non-Black infants, the combination ELBW/PostTD had two mortalities among 25 births (r/n = 2/25). The same combination (ELBW/PostTD) was observed for Black infants with zero mortalities among six births (r/n = 0/6). For Black infants, ELBW/TD had three mortalities among 52 births (r/n = 3/52); VLBW/PostTD had zero mortalities among 14 births (r/n = 0/14); and NBW/EPTD had two mortalities among 74 births (r/n = 2/74).

Table 2 lists the POs for combinations of BW and LG. The risk of infant mortality increased 100-fold between the normal combination (NBW/TD; PO < 0.004) and the smallest BW and earliest LG (ELBW/EPTD; PO > 0.4). The more rarely observed combinations (see above) had relatively wide confidence intervals.

The relative risks for incidences of birth conditions are listed in Table 2. Black infants were at increased risk of smaller and earlier preterm births. The racial relative risk (Black vs. non-Black) was 2.76 (2.72, 2.79) for ELBW/EPTD.

The conditional risk for infant mortality (likelihood of infant mortality for the specific birth condition) was higher for Black infants with normal BW and term or post-term delivery (NBW/PTD and NBW/PostTD). However, there were multiple combinations for which a higher conditional mortality rate was observed among non-Black infants (Table 2).

Estimates of PA showed that the combination ELBW/EPTD explained 61.4% (60.3, 62.4) of the racial disparity. One other combination, LBW/PTD, explained 4.0% (1.3, 6.5), while all other combinations had confidence intervals for PA that included zero (Table 2).

For objective 2, results for the computation of PA for the combinations of ELBW/EPTD and LBW/PTD are presented in Table 3.

Evaluation of sensitivity to the priors showed the PO estimates were lower with the beta(1,10) prior than the uniform(0,1) prior but only for the rarest combinations. For common combinations of BW/LG, the posterior predictions were similar for both priors. The estimates for PA were robust with respect to the choice of priors. Results for Model 2 with the uniform(0,1) priors are listed in Appendix B.

## 4. Discussion

The results motivating the current study showed that approximately 75% of racial disparity in infant mortality in the U.S. could be prevented by normalizing BWs [3]. The current study confirms that the prediction that eliminating abnormal BWs reduces racial disparity by approximately 75% was still valid when controlling for both confounding and interaction with abnormal LGs. The current study showed that when BWs were normal, infant mortality was both relatively low and relatively similar between race groups across categories of LG. It was also true that stabilizing all deliveries as term deliveries would have the same characteristics: relatively low infant mortality rates that are similar for different race groups across multiple BW categories.

The second objective was to parse these overall effects of BW and LG into the portions that could be attributed to each specific combination of the two five-category mediators. Estimation of PA for the control of a single combination of two multi-category mediators was more complicated than estimating PA with a single binary mediator and required multiple steps. Each step provided Bayesian estimates of the full posterior distribution. The steps are described as Equations (5)–(9) in both the Methods section and the MultiBUGS code provided in the Appendix. The first step estimated the number of cases of infant mortality, for both race groups, under the control strategy for a specific combination of BW/LG (Equation (5)). The second step estimated the new number of cases of infant mortality, for both race groups in the population (Equation (6)). The third step estimated the new PO by race (Equation (7)). The CDE was the difference in the new race rates (Equation (8)). The PA used the standard equation (Equation (9)). In addition to the large contribution to racial disparity of the ELBW/EPTD combination, there was a smaller but significant contribution to racial disparity by the much more common combination of LBW/PTD. The interaction between two multi-category mediators modeled in this study provides important insight into the contributing factors for racial disparity.

It has been claimed that individual risk factors alone have not explained the persistent racial disparity [2]. If true, this would provide a challenge to mediation modelers. Both model-based counterfactual mediation analysis and Bayesian PO analysis rely on personal risk inference. Contemporary mediation modeling incorporates counterfactual logic by following the long-held view that causal effects must be defined at the individual level. Because an individual can only have a single outcome at any one time, investigating individual-level causal effects has been referred to as the fundamental problem of causal inference [9]. The circumvention under a counterfactual approach estimates the population-average causal effects and applies the average as an individual’s expectation. To ensure population-level mediation estimates have a causal interpretation for individuals, four assumptions on potential confounding need to hold [7]. A direct Bayesian approach that does not include counterfactual considerations was proposed long ago [10]. In Bayesian modeling, the condition of disease versus non-disease for an individual is the product of a probability process. It is the probability of the process, not the factual outcome, that is important in the analysis. This probability is best called the PO. Conditional upon the assumption that the model is correct and uncertainty that is estimable, all patients with a matching vector of causes have the same probability of disease or PO.

Instead of personal risk, reviewers have reasoned that we must look to social determinants as causes [1,2]. Social determinants include economic stability, education, healthcare access and quality, neighborhood and built environment, and social and community context [1,2]. The reviews identify two correlations, first between minority populations and these social determinants and second between the social determinants and racial disparities, including infant mortality. These associations do not appear to have been formally linked by mediation modeling. Community level factors as claimed causes should not deter mediation modeling. Community and living location factors are readily interpretable as individual risks and amenable to mediation modeling by Bayesian spatial modeling, for example [11].

The modeling of mediation for the multivariate interaction node, in this study, is a novel approach. Expanding a classical approach from the two-category mediator to a 25-level multivariate variable would be extremely challenging. The regression or model-based approach has been the predominant approach for models with binary causes and binary outcomes [12,13,14,15,16]. The first step in a model-based approach is to estimate regression parameters for a set of equations, with one of the equations regressing causes, including the interaction on the logit transform of the binomial outcome. Similar regression modeling would be possible by coding all possible interactions with dummy variables. The POs for each combination of cause and mediator could be estimated from the reverse transform of the product from the regression equations. This would take a matrix of binary dummy variables and many regression coefficients but nevertheless could estimate the PO for each combination of race, BW, and LG that was observed. The Bayesian approach to estimate PO, reported here, is much simpler and is the fundamental Bayesian estimation of probability from counts of birth and infant mortality.

### Limitations

The validity for each specific PO is based upon three assumptions [4]. The first assumption is the Stable Unit Treatment Value Assumption (SUTV). Rubin describes two parts. The first is consistency where it was assumed that the birthweights were well defined and the PO was independent of how the birthweights were assigned. The second part of the SUTVA assumption was that there was no correlation among infants. The second part may have been violated in that many women will have had multiple infants over the three-year study period. Within the cluster known as her “family”, children could have had correlation among birthweights and correlated outcomes for IM. In the situation where cluster size (family) was small relative to the sample of women, the effect will have been quite small. The second assumption is positivity, where each infant was assumed to have had some non-zero probability for the classification of BW. The third assumption was that BW assignments were random, conditional upon the additional variables in the model. This condition can be understood simply as there are no influential causes missing from the model. The prior causal inference that 75% of this racial disparity could have been mediated by normalizing all birthweights raised the question: could the relationship more accurately be attributed to premature births? This question raises concern for possible violation of the “no missing variables” assumption. The question of how including preterm birth modifies the causal inference regarding BW and IM should be addressed.

Limitations for this study include the likelihood of missing mediators that could contribute meaningfully to the estimation of racial disparity. The outcome-based mediation model implemented here controlled confounding and interaction by stratification [17]. This control is provided by estimating the PO for every combination of variables in the model. Any criticism for possible missing confounders should claim that the missing factor confounds the estimation of racial disparity, not the outcome of IM. Further testing should be performed by adding potential mediators into the interaction. The current study is proposing a very specific biologic process as contributary to racial disparity, but it remains yet to be determined how social determinants [1,2] may confound this inference. An abbreviated view of the current findings is that extreme BW and LG (EBWLG) was contributory to racial disparity for IM. The evaluation of a social determinant like a specific level of education (ED), for example, would require the estimation of racial difference in POs among levels of EBWLG crossed with ED. The bases for prior beliefs about confounders for racial disparity are relatively complex but the testing as illustrated in the current study is straightforward.

As more effects are added to the interaction model, the possibility of sparse cells of data becomes a limitation and PO estimates can become imprecise. The study provided two responses to this limitation. First, some combinations were not observed among more than 25 million births. The best approach for these combinations was to report that they did not occur. For the combinations that were observed, sensitivity to the specified priors was evaluated, and the POs for rare combinations (e.g., very small infants born post-term) varied with the choice of priors. The two priors that were compared were the beta(1,10) and the uniform(0,1) priors. Both priors are considered minimally informative, but no prior can be uninformative. The beta(1,10) prior favors POs likely to be near zero, and the uniform(0,1) prior provides equal prior likelihood for all values from zero to one. For the rarely observed combinations of risks, the estimate of PO was lower for the estimates with beta(1,10) prior than for the uniform(0,1) prior. However, for most combinations, there were sufficient observations that estimates for POs using either prior were very similar. In contrast to the POs (potential outcomes), the PA (percentage attributable) were robust with respect to the choice of priors. This can be expected with the modeling approach that was used because the PA estimates for each combination do not use the POs for the combination, but they do use the POs for the combination used as the control strategy. This can be seen in Equations (5)–(9). In this study, the important PO was PO [race,NBW,TD] which was both precise and robust with respect to priors. The prediction that controlling a very rare combination of risks will not have a substantial public health impact should be considered reliable. In further studies using this study’s novel approach, investigators will need to address sparse cells. As an alternative, going forward, complex interactions can be simplified and studied in partitions. It would be reasonable to investigate further mediators within a specific stratum from the current study. For example, the mediation of EPTD/ELBW (a single stratum in this study) can be studied alone. This would logically compare the racial characteristics of infants born ELBW/EPTD relative to NBW/TD.

How race and racial disparity are defined also presents limitations. Using the mother’s race designation does provide a misrepresentation of an infant’s race when the father’s binary designation was the alternative race grouping. However, the mother’s identity is the preferred classification because it is known with certainty and the father’s is often not identified. As an additional limitation, this study combines multiple subgroups into both Black and non-Black groups. While considerable information can be gleaned with more specific race definitions [18], this study has focused on the common definition and the evaluation of racial disparity of most public interest. More study involving specific race groups as potential subjects of racial disparity is encouraged. However, in the evaluation of racial disparities, the pooling of all race groups or all other race groups as the referent group, as opposed to pair-wise comparisons, has been recommended [19].

## 5. Conclusions

The current study provided a motivating example identifying individual risk and illustrates how Bayesian estimation of POs enables complex mediational investigations. Bayesian methodology designed to model a complex multivariate interaction node, simple Bayesian estimation of POs, and a combination of estimates using Markov Chain Monte Carlo have promising potential for further investigation.

## Figures and Tables

**Table 1:** Mediational effects for abnormal length of gestation or birthweight.

Mediator	Controlled Direct Effect	Mediated Effect	Percent Attributable
Birthweight	0.0011 (0.0010, 0.0012)	0.0033 (0.0032, 0.0034)	74.6 (72.7, 76.4)
Length of gestation	0.0013 (0.0012, 0.0013)	0.0032 (0.0030, 0.0033)	71.7 (69.7, 73.6)
Birthweight and length of gestation	0.0011 (0.0010, 0.0012)	0.0033 (0.0032, 0.0034)	74.9 (73.0, 76.8)

**Table 2:** Results for risks for combinations of five birthweight and five length of gestation categories using a beta(1,10) prior for potential outcomes.

Birthweight	Length of Gestation	Potential Outcome (Infant Mortality)		Racial Relative Risk (Black vs. Non-Black) (Black Versus Non-Black Infant)		Percentage Attributable
		Black	Non-Black	Rate of Conditions	Conditional Rate of Mortality	
ELBW	EPTD	0.410 (0.404, 0.416)	0.417 (0.414, 0.420)	2.75 (2.72, 2.79)	0.98 (0.97, 1.00)	61.4 (60.3, 62.4)
ELBW	VPTD	0.084 (0.076, 0.093)	0.094 (0.089, 0.098)	2.44 (2.36, 2.53)	0.90 (0.80, 1.00)	1.74 (−0.95, 4.41)
ELBW	PTD	0.175 (0.131, 0.225)	0.201 (0.179, 0.225)	1.95 (1.68, 2.23)	0.87 (0.64, 1.15)	0.16 (−2.59, 2.88)
ELBW	TD	0.059 (0.018, 0.135)	0.069 (0.045, 0.100)	1.61 (1.19, 2.13)	0.86 (0.25, 2.22)	0.00 (−2.74, 2.73)
ELBW	PostTD	0.043 (0.002, 0.207)	0.076 (0.018, 0.194)	2.41 (0.94, 5.34)	0.56 (0.02, 4.81)	0.00 (−2.75, 2.72)
VLBW	EPTD	0.083 (0.074, 0.093)	0.098 (0.094, 0.103)	1.88 (1.81, 1.95)	0.85 (0.74, 0.96)	0.86 (−1.87, 3.55)
VLBW	VPTD	0.037 (0.034, 0.040)	0.043 (0.041, 0.044)	2.21 (2.18, 2.25)	0.87 (0.80, 0.95)	2.40 (−0.29, 5.07)
VLBW	PTD	0.041 (0.037, 0.047)	0.058 (0.055, 0.061)	2.06 (2.00, 2.11)	0.72 (0.63, 0.81)	0.65 (−2.10, 3.33)
VLBW	TD	0.084 (0.063, 0.110)	0.088 (0.079, 0.098)	1.68 (1.54, 1.84)	0.96 (0.70, 1.27)	0.16 (−2.59, 2.87)
VLBW	PostTD	0.028 (0.001, 0.143)	0.044 (0.017, 0.089)	1.15 (0.64, 1.89)	0.63 (0.02, 4.22)	−0.01 (−2.76, 2.72)
LBW	EPTD	0.161 (0.116, 0.214)	0.223 (0.198, 0.250)	1.91 (1.64, 2.21)	0.72 (0.51, 0.99)	0.08 (−2.66, 2.80)
LBW	VPTD	0.038 (0.035, 0.042)	0.039 (0.037, 0.040)	1.48 (1.45, 1.51)	0.99 (0.88, 1.10)	0.89 (−1.85, 3.58)
LBW	PTD	0.014 (0.013, 0.015)	0.014 (0.014, 0.014)	1.65 (1.63, 1.66)	1.00 (0.95, 1.06)	3.96 (1.28, 6.58)
LBW	TD	0.011 (0.010, 0.012)	0.012 (0.011, 0.012)	1.78 (1.76, 1.79)	0.94 (0.88, 1.00)	2.18 (−0.54, 4.85)
LBW	PostTD	0.042 (0.029, 0.059)	0.034 (0.028, 0.040)	1.71 (1.58, 1.86)	1.25 (0.82, 1.82)	0.13 (−2.62, 2.85)
NBW	EPTD	0.032 (0.008, 0.083)	0.050 (0.034, 0.072)	1.45 (1.12, 1.83)	0.63 (0.14, 1.78)	−0.01 (−2.76, 2.72)
NBW	VPTD	0.029 (0.019, 0.043)	0.047 (0.041, 0.052)	1.27 (1.18, 1.37)	0.63 (0.40, 0.95)	−0.06 (−2.82, 2.66)
NBW	PTD	0.007 (0.007, 0.008)	0.006 (0.006, 0.006)	1.04 (1.03, 1.05)	1.27 (1.18, 1.37)	0.57 (−2.19, 3.27)
NBW	TD	0.003 (0.003, 0.003)	0.002 (0.002, 0.002)	0.98 (0.98, 0.98)	1.66 (1.61, 1.71)	NA
NBW	PostTD	0.002 (0.002, 0.003)	0.001 (0.001, 0.002)	0.96 (0.95, 0.96)	1.57 (1.37, 1.78)	−0.27 (−3.05, 2.48)
MAC	EPTD	NA	NA	NA	NA	NA
MAC	VPTD	NA	NA	NA	NA	NA
MAC	PTD	0.015 (0.010, 0.022)	0.013 (0.011, 0.014)	0.83 (0.79, 0.88)	1.22 (0.78, 1.85)	−0.01 (−2.76, 2.72)
MAC	TD	0.002 (0.002, 0.002)	0.001 (0.001, 0.001)	0.57 (0.57, 0.58)	1.79 (1.55, 2.06)	0.18 (−2.60, 2.91)
MAC	PostTD	0.002 (0.001, 0.002)	0.001 (0.001, 0.001)	0.59 (0.58, 0.60)	1.50 (0.99, 2.18)	−0.02 (−2.76, 2.69)

**Table 3:** Study results in the estimation of percentage of attributable effect.

		ELBW/EPTD		LBW/PTD	
		Black	Non-Black	Black	Non-Black
Count of births in stratum (from data)		26,163	88,158	114,701	647,261
Current count of infants in stratum (from data)		10,729	36,779	1614	9088
New count in stratum (from Equation (5))		73 (57, 90)	148 (124, 172)	320 (284, 356)	1085 (1020, 1150)
New count of infant mortality in population (from Equation (6))		11,230 (11,210, 11,240)	65,010 (64,990, 65,030)	20,590 (20,550, 20,620)	93,640 (93,570, 93,700)
New racial PO in population (from Equation (7))		0.0045 (0.0045, 0.0045)	0.0028 (0.0028, 0.0028)	0.0083 (0.0083, 0.0083)	0.0041 (0.0041, 0.0041)
Controlled direct effect (CDE) (from Equation (8))		0.0017 (0.0017, 0.0017)		0.0042 (0.0042, 0.0043)	
Percentage attributable (PA) (from Equation (9))		61.4 (60.3, 62.4)		4.0 (1.3, 6.6)	

## Data Availability

The data for this study is provided in Appendix A.

## Appendix A. MultiBUGS Code and Data

model{for (i in 1:50){ r[i] ~ dbin (PO[R[i],BW[i],LG[i]],n[i])

           mv.r[R[i],BW[i],LG[i]] < − r [i]

           mv.n[R[i],BW[i],LG[i]] < − n [i] }

for (a in 1:2){for (b in 1:5){for (c in 1:5){   PO[a,b,c] ~ dbeta(1,10)

                           # PO[a,b,c] ~ dunif(0,1)

                           mv.n[a,b,c] ~ dbin (rf [a,b,c], sum.n.race [a])

                           rf[a,b,c] ~ dunif(0,1) }}}

for (b in 1:5){for (c in 1:5){     RR.factor[b,c] < − rf [2,b,c]/rf [1,b,c]

                        RR.race[b,c] < − PO [2,b,c]/PO [1,b,c]} }

for (a in 1:2){for (i in 1:50){     r.race[i,a] < − equals (R[i],a) * r[i] n.race[i,a] < −equals (R[i],a) * n [i]

     }

                         sum.n.race[a] < − sum(n.race[,a])

                         sum.r.race[a] < − sum(r.race[,a])

                         sum.r.race[a] ~ dbin (race.PO[a], sum.n.race[a])

                         race.PO[a] ~ dunif(0,1) }

TE < − race.PO[2] − race.PO[1]	
RR < − race.PO[2]/race.PO[1]	
# Equations (5)–(9) are looped twice to avoid strata with *n* = 0	
for (b in 1:4){for (c in 1:5){ for (a in 1:2){	
s.r[a,b,c] ~ dbin(PO[a,4,4], mv.n[a,b,c])	#equation 5
p.r[a,b,c] < − sum.r.race[a] − (mv.r[a,b,c]-s.r[a,b,c])	#equation 6
new.PO[a,b,c] < − p.r[a,b,c]/sum.n.race[a]}	#equation 7
CDE[b,c] < − new.PO[2,b,c] − new.PO[1,b,c]	#equation 8
PA[b,c] < − 100 * (TE − CDE[b,c])/TE }}	#equation 9
for (b in 5:5){for (c in 3:5){ for (a in 1:2){	
s.r[a,b,c] ~ dbin(PO[a,4,4], mv.n[a,b,c])	#equation 5
p.r[a,b,c] < − sum.r.race[a] − (mv.r[a,b,c]-s.r[a,b,c])	#equation 6
new.PO[a,b,c] < − p.r[a,b,c]/sum.n.race[a]}	#equation 7
CDE[b,c] < − new.PO[2,b,c] − new.PO[1,b,c]	#equation 8
PA[b,c] < − 100 × (TE – CDE[b,c])/TE }} }	#equation 9
	

data

R = 1 is non-Black

R = 2 is Black

BW = 1 is extremely low BW (ELBW; <1.0 kg);

BW = 2 is very low BW (VLBW; 1.0 kg to 1.5 kg);

BW = 3 is low BW (LBW; 1.5 kg to 2.5 kg);

BW = 4 is normal BW (NBW; 2.5 kg to 4.0 kg);

BW = 5 is macrosomia (MAC; >4.0 kg).

LG = 1 is extremely preterm delivery (EPTD; <28 weeks);

LG = 2 is very preterm delivery (VPTD; 28 to 32 weeks);

LG = 3 is preterm delivery (PTD; 32 to 37 weeks);

LG = 4 is term delivery (TD; 37 to 41 weeks);

LG = 5 is post-term delivery (PostTD; > 41weeks).

R[]	BW[]	LG[]	n[]	r[]
1	1	1	88,158	36,779
1	1	2	16,371	1537
1	1	3	1145	232
1	1	4	304	21
1	1	5	25	2
1	2	1	15,756	1552
1	2	2	69,530	2973
1	2	3	27,906	1616
1	2	4	3069	271
1	2	5	118	5
1	3	1	1002	225
1	3	2	58,717	2277
1	3	3	647,261	9088
1	3	4	496,291	5788
1	3	5	3618	123
1	4	1	479	24
1	4	2	5552	259
1	4	3	897,770	5192
1	4	4	17,660,709	29,602
1	4	5	1,103,035	1636
1	5	1	0	0
1	5	2	0	0
1	5	3	16,498	206
1	5	4	1,592,318	1943
1	5	5	267,424	290
2	1	1	26,163	10,729
2	1	2	4308	364
2	1	3	239	43
2	1	4	52	3
2	1	5	6	0
2	2	1	3191	266
2	2	2	16,576	616
2	2	3	6188	256
2	2	4	556	47
2	2	5	14	0
2	3	1	205	34
2	3	2	9365	358
2	3	3	114,701	1614
2	3	4	94,945	1037
2	3	5	666	28
2	4	1	74	2
2	4	2	758	22
2	4	3	100,501	738
2	4	4	1,865,735	5196
2	4	5	113,934	264
2	5	1	0	0
2	5	2	0	0
2	5	3	1482	22
2	5	4	98,558	215
2	5	5	16,944	27
END				

## Appendix B. Study Results with Uniform(0,1) Prior for Potential Outcomes

**Table:**

Birthweight	Length of Gestation	Potential Outcome (Infant Mortality)		Racial Relative Risk (Black vs. Non-Black) (Black Versus Non-Black Infant)		Percentage Attributable
		Black	Non-Black	Rate of Conditions	Conditional Rate of Mortality	
ELBW	EPTD	0.410 (0.404, 0.416)	0.417 (0.414, 0.420)	2.76 (2.72, 2.79)	0.98 (0.97, 1.00)	61.4 (60.3, 62.4)
ELBW	VPTD	0.085 (0.077, 0.093)	0.094 (0.090, 0.098)	2.44 (2.36, 2.53)	0.90 (0.81, 1.01)	1.76 (−0.97, 4.34)
ELBW	PTD	0.182 (0.136, 0.233)	0.203 (0.180, 0.227)	1.94 (1.69, 2.23)	0.90 (0.66, 1.18)	0.18 (−2.61, 2.80)
ELBW	TD	0.069 (0.021, 0.156)	0.071 (0.046, 0.103)	1.61 (1.19, 2.14)	0.97 (0.28, 2.51)	0.03 (−2.77, 2.65)
ELBW	PostTD	0.093 (0.003, 0.403)	0.102 (0.025, 0.250)	2.42 (0.95, 5.38)	0.91 (0.03, 7.14)	0.02 (−2.78, 2.64)
VLBW	EPTD	0.084 (0.074, 0.093)	0.099 (0.094, 0.103)	1.88 (1.81, 1.95)	0.85 (0.75, 0.96)	0.88 (−1.90, 3.50)
VLBW	VPTD	0.037 (0.034, 0.040)	0.043 (0.041, 0.044)	2.21 (2.18, 2.25)	0.87 (0.80, 0.95)	2.42 (−0.31, 5.00)
VLBW	PTD	0.041 (0.037, 0.047)	0.058 (0.055, 0.061)	2.06 (2.00, 2.12)	0.72 (0.63, 0.81)	0.66 (−2.11, 3.27)
VLBW	TD	0.085 (0.064, 0.110)	0.089 (0.079, 0.099)	1.69 (1.53, 1.84)	0.97 (0.71, 1.28)	0.18 (−2.62, 2.80)
VLBW	PostTD	0.045 (0.002, 0.220)	0.047 (0.019, 0.096)	1.15 (0.63, 1.92)	0.95 (0.03, 6.14)	0.02 (−2.79, 2.64)
LBW	EPTD	0.168 (0.120, 0.222)	0.225 (0.200, 0.252)	1.91 (1.63, 2.21)	0.75 (0.52, 1.01)	0.11 (−2.69, 2.73)
LBW	VPTD	0.038 (0.035, 0.042)	0.039 (0.037, 0.040)	1.48 (1.45, 1.51)	0.99 (0.88, 1.10)	0.92 (−1.84, 3.53)
LBW	PTD	0.014 (0.013, 0.015)	0.014 (0.014, 0.014)	1.65 (1.64, 1.66)	1.00 (0.95, 1.06)	3.98 (1.27, 6.53)
LBW	TD	0.011 (0.010, 0.012)	0.012 (0.011, 0.012)	1.78 (1.76, 1.79)	0.94 (0.88, 1.00)	2.20 (−0.56, 4.85)
LBW	PostTD	0.043 (0.029, 0.060)	0.034 (0.029, 0.041)	1.71 (1.58, 1.85)	1.25 (0.83, 1.83)	0.15 (−2.64, 2.77)
NBW	EPTD	0.036 (0.008, 0.093)	0.051 (0.034, 0.073)	1.45 (1.12, 1.83)	0.70 (0.16, 1.98)	0.02 (−2.79, 2.64)
NBW	VPTD	0.030 (0.019, 0.044)	0.047 (0.042, 0.053)	1.27 (1.18, 1.37)	0.64 (0.41, 0.95)	−0.04 (−2.84, 2.59)
NBW	PTD	0.007 (0.007, 0.008)	0.006 (0.006, 0.006)	1.04 (1.03, 1.05)	1.27 (1.18, 1.37)	0.58 (−2.22, 3.27)
NBW	TD	0.003 (0.003, 0.003)	0.002 (0.002, 0.002)	0.98 (0.98, 0.98)	1.66 (1.61, 1.71)	NA
NBW	PostTD	0.002 (0.002, 0.003)	0.001 (0.001, 0.002)	0.96 (0.95, 0.96)	1.57 (1.37, 1.78)	−0.25 (−3.06, 2.43)
MAC	EPTD	NA	NA	NA	NA	NA
MAC	VPTD	NA	NA	NA	NA	NA
MAC	PTD	0.015 (0.010, 0.022)	0.013 (0.011, 0.014)	0.83 (0.79, 0.88)	1.22 (0.77, 1.82)	0.01 (−2.78, 2.64)
MAC	TD	0.002 (0.002, 0.002)	0.001 (0.001, 0.001)	0.57 (0.57, 0.58)	1.79 (1.55, 2.07)	0.18 (−2.60, 2.85)
MAC	PostTD	0.002 (0.001, 0.002)	0.001 (0.001, 0.001)	0.59 (0.58, 0.60)	1.50 (1.00, 2.17)	0.00 (−2.80, 2.63)
